# Enhanced metabolic activities for ATP production and elevated metabolic flux via pentose phosphate pathway contribute for better CIK cells expansion

**DOI:** 10.1111/cpr.12594

**Published:** 2019-03-07

**Authors:** Weiwei Zhang, Huimin Huang, Haibo Cai, Wen‐Song Tan

**Affiliations:** ^1^ State Key Laboratory of Bioreactor Engineering East China University of Science and Technology Shanghai China

**Keywords:** CIK cells, energy metabolism, ex vivo expansion, glucose and glutamine metabolism, oxygen transfer

## Abstract

**Objective:**

Ex vivo expansion is an effective way to produce cytokine‐induced killer (CIK) cells needed for clinical trials. Here, ex vivo expansion and metabolism characters of CIK cells in static and dynamic cultures and the relationship between cell expansion and metabolism were investigated.

**Materials and methods:**

Oxygen transfer efficiency was assessed by computational fluid dynamics technique. Cell phenotype, apoptosis and of transporter expression were determined by flow cytometry and Western blotting. Metabolites and enzyme activities were assessed by biochemical methods.

**Results:**

Dynamic cultures favoured better CIK cell expansion without impairing their phenotype and cytotoxicity, enhanced oxygen transfer efficiency. The glucose metabolism flux of cells in dynamic cultures was enhanced by upregulating surface glucose transporter 1 expression and phosphofructokinase activity. Moreover, pentose phosphate pathway (PPP) metabolic flux was enhanced through upregulating glucose‐6‐phosphate dehydrogenase activity. Glutaminolysis was also accelerated via boosting glutamine transporters expression, glutaminase (GLS) and glutamate dehydrogenase activities. Together with higher oxygen consumption rate and extracellular acidification rate, it was suggested that cells in dynamic cultures were in a more vigorous metabolic state for ATP production.

**Conclusion:**

Dynamic cultures accelerated glucose and glutamine metabolic flux to promote ATP production, elevated glucose metabolic flux through PPP to promote biosynthesis for better cell expansion. These findings may provide the basis for ex vivo CIK cell expansion process optimization.

## 1 INTRODUCTION

Cancer is among the top killer diseases and acts as the main cause of the morbidity and mortality according to the global and Chinese cancer statistics.^1^, ^2^ Adoptive cellular therapy raises growing interest and holds great promise in treating various malignant tumours. CIK cells are considered as one of the ideal candidate cell types for cancer immunotherapy,^3^, ^4^, ^5^ as they can recognize and destroy tumour cells effectively in a human leucocyte antigen‐independent way.^6^, ^7^, ^8^

Traditionally, CIK cells are generated from mononuclear cells ex vivo by initial priming with interferon‐γ (IFN‐γ) and anti‐CD3 antibody followed by repeated stimulation with interleukin‐2 (IL‐2) for 2 weeks.^9^ The obtained CIK cells are heterogeneous lymphocytes, in which over 90% are CD3^+^ cells, and can be divided into two main subsets: CD3^+^ CD56^+^ cells and CD3^+^ CD56^‐^ T cells.^10^, ^11^, ^12^, ^13^ Due to the limited initial number, CIK cells need to be expanded ex vivo up to 10^10^ cells per infusion to meet the clinical requirements.^14^, ^15^, ^16^ Moreover, it was reported that the clinical response was related to injected cell numbers^17^, ^18^, ^19^; therefore, it is necessary to optimize the ex vivo CIK cells expansion process for better clinical efficacy.

For proliferation, CD3^+^ cells need to enter into an activation state from a quiescent state, because only cells which were fully activated could undergo cell proliferation.^20^ The activation process required the production and surface expression of nutrient transporters, co‐stimulatory molecules, etc, all of which require energy. Besides the large energy burden after CD3^+^ cells were activated, there was also an increasing demand for biosynthetic precursor molecules,^21^ which was also an energy‐consuming process.^22^ Therefore, it is important to provide sufficient energy and promote the biosynthesis of lipids, proteins, nucleic acids and other carbohydrates for the proliferation of CD3^+^ cells.

Glucose is one of the most important carbon and energy source for CD3^+^ cells. The uptake of glucose in CD3^+^ cells is mainly via glucose transporter 1 (GLUT1).^23^, ^24^, ^25^ After entering cells, glucose is phosphorylated to glucose‐6‐phosphate (G6P). G6P could be converted into pyruvate by phosphofructokinase (PFK), pyruvate kinase, etc through glycolysis, it also could be converted to ribose‐5‐phosphate by glucose‐6‐phosphate dehydrogenase (G6PDH), etc through pentose phosphate pathway (PPP). Glucose‐derived pyruvate either entered into tricarboxylic acid (TCA) cycle or was converted to lactate by lactic dehydrogenase. High‐glucose metabolic flux rate allowed rapid macromolecular synthesis and ATP generation, which were all necessary for cell proliferation.^23^, ^26^ It had been reported that an almost 20‐fold increase was induced in glucose metabolic flux of thymocytes after activation.^23^ In addition, glucose deficiency would inhibit T‐cell proliferation and survival.^24^

In addition to glucose, glutamine is also an important energy source. In human CD3^+^ cells, there are several well‐characterized transporters, such as ASC amino acid transporter 2 (ASCT2), sodium‐coupled neutral amino acid transporter 1/2 (SNAT1/SNAT2) and a heterodimer transporter (CD98/large neutral amino acid transporter, LAT1).^27^, ^28^, ^29^ After entering cells, glutamine could be converted to α‐ketoglutarate by glutaminase (GLS), glutamate dehydrogenase (GDH), etc for TCA cycle replenishment and oxidative phosphorylation (OXPHOS) to produce metabolic intermediates and ATP for cell proliferation.^30^, ^31^ Evidence was obtained that glutamine consumption was enhanced after the activation of CD3^+^ cells.^32^ It had also been indicated that T cells were unable to proliferate in glutamine‐free medium.^28^ Thus, it was necessary to monitor glucose and glutamine concentrations in culture environment and promote their metabolic rates for better cell proliferation during the ex vivo expansion of CIK cells.

For ATP productionvia OXPHOS, oxygen is one of the indispensable factors. It was illustrated that the oxygen consumption rate of activated CD3^+^cells was doubled as that of quiescent CD3^+^ cells.^26^, ^33^ Moreover, cell metabolism patterns were closely associated with oxygen.^34^, ^35^ Das et al^36^ showed that hyperoxia decreased both glycolytic and OXPHOS capacity of MLE‐12 cells, while Goto et al^37^ found that hypoxic changed the metabolism of THP‐1 cells from OXPHOS to glycolysis. Therefore, improving oxygen supply may also be beneficial for better cell proliferation during the ex vivo expansion of CIK cells.

The conventional ex vivo expansion of CIK cells was carried out mainly in static cultures with gas‐permeable culture bags or T‐flasks.^38^, ^39^, ^40^ In static cultures, cells sank to the bottom of culture bags or flasks, the microenvironment surrounding cells was heterogeneous because of lacking mixing.^41^, ^42^ Nevertheless, this environmental inhomogeneity could be overcome by dynamic cultures. It was reported that dynamic cultures could promote the ex vivo expansion of immune effector cells. For instance, Bohnenkamp et al^43^ found more CD3^+^ T cells were expanded in dynamic cultures using a stirred bioreactor than in static cultures using T‐flasks. In line with this, Donia et al^44^ illustrated a 4‐times higher expansion fold of T cells in dynamic cultures using wave bioreactors than in static cultures using bags. In our previous study, a dynamic culture was adopted in order to optimize the ex vivo expansion of CIK cells and found that dynamic cultures could support effective cell expansion of CIK cells and maintain their physiological function.^45^ However, the relationship between cell metabolism and expansion remains unknown.

Thus, with the objective to understand the mechanism how dynamic cultures affect cell expansion, in this work, the computational fluid dynamics (CFD) technique was first used to show the oxygen transfer difference between static and dynamic cultures. Furthermore, the glucose, glutamine and energy metabolism of CIK cells in static and dynamic cultures was elucidated by the analysis of nutrient transporters expression, key enzymes activities and nutrients consumption. Specifically, two ATP‐generating pathways, glycolysis and OXPHOS, were analysed to evaluate the ATP production ability of cells in static and dynamic cultures. These findings would provide the basis for optimization of ex vivo CIK or other immune effector cells expansion.

## 2 MATERIALS AND METHODS

### 2.1 Cell preparation

The experiments conducted in this study were approved by the Science Ethics committee of the State Key Laboratory of Bioreactor Engineering, East China University of Science and Technology and were in accordance with the guidelines for cellular products research and preparation, China (2016). CIK cells were generated from cord blood mononuclear cells (CBMNCs) of full‐term healthy delivery with informed consent. CBMNCs were enriched using density gradient centrifugation on Ficoll/Histopaque (density: 1.077 g/mL; GE Healthcare, New York, NY, USA) and cultured in static and dynamic cultures for the generation of CIK cells as our previous study.^45^ Culture supernatants were mixed sufficiently before sampling. Cell numbers were counted every day.

As indicated assays, CD3^+^ cells were isolated from fresh CBMNC using a CD3 antibody‐conjugated paramagnetic microbeads and MiniMACS columns (Miltenyi Biotech, Bergisch Gladbach, Germany). The purity of fresh isolated CD3^+^ cells was over 90% as assessed by flow cytometry.

### 2.2 Cell apoptosis

Cell apoptosis was detected by using Alexa Fluor^®^ 488 Annexin V/Dead Cell Apoptosis Kit (Life Technologies, Waltham, MA, USA) according to the manufacturer's instructions. The fraction of apoptotic cells in cell preparations was analysed by flow cytometry.

### 2.3 Cell phenotype

A total of 1 × 10^6^ cells were stained with CD3‐FITC, CD56‐PE, CD8‐Percp‐cy5.5 and/or CD4‐APC mouse anti‐human CD4 antibodies. Cell preparations were analysed on a flow cytometer (FACS Aria I; BD Bioscience, San Jose, CA, USA) to determine the proportions of CD3^+^ cells, CD3^+^CD56^+^ cells, CD3^+^CD8^+^ cells and CD3^+^CD4^+^ cells in total cell population. All antibodies were purchases from BD bioscience.

### 2.4 Physiological function assays of expanded CIK cells

Physiological function of expanded CIK cells can be evaluated by its ex vivo cytotoxic capacity on tumour cells, the expression of CD107a and intracellular expression of cytotoxic granule proteins.

The cytotoxic capacity of expanded CIK cells on K562, MB231, 22Rv1, DU145, HeLa and HepG2 cells was assessed with Cell Counting Kit‐8 (Dojindo) according to the manufacturer's instructions as our previous study.^45^

For cell degranulation, CIK cells were co‐cultured with K562 cells at an E:T ratio of 10:1 for 4 hours and then stained with CD3‐FITC, CD56‐PE, CD8‐PerCP‐Cy5.5 and CD107a‐PE‐Cy7 mouse anti‐human antibodies (BD Bioscience) for analysing the CD107a expression of CD3^+^ cells, CD3^+^CD56^+^ cells, CD3^+^CD8^+^ cells.

To carry out the intracellular perforin and granzyme B (Gz‐B) of cells in cultures, cells were stained with surface markers (CD3‐FITC, CD56‐PE and CD8‐PerCP‐Cy5.5), then fixed, permeabilized and stained with antibodies of BV421‐perforin (BD Bioscience) and V450‐granzyme B (BD Bioscience) for analysing intracellular perforin and Gz‐B expression of CD3^+^ cells, CD3^+^CD56^+^ cells and CD3^+^CD8^+^ cells.

### 2.5 Glucose uptake activity

Glucose uptake activity was measured by a fluorescent D‐glucose analogue 2‐(*N*‐(7‐nitrobenz‐2‐oxa‐1,3‐diaz‐ol‐4‐yl) amino)‐2‐deoxy‐d‐glucose (2‐NBDG; Thermofisher, Waltham, MA, USA) assay. Briefly, 1 × 10^6^ cells were collected and washed, then incubated with 200 μmol/L 2‐NBDG for 1 hour at 37°C in a humidified incubator with 5% CO_2_. After incubation, the mean fluorescence intensity (MFI) of intracellular 2‐NBDG was immediately measured using a ImageStream^X^ Mark II imaging flow cytometer on FITC channel (Merck, Darmstadt, Germany).

### 2.6 Analysis of glutamine and ammonia concentrations in culture supernatant

Glutamine and ammonia concentrations in culture supernatants were analysed using a Nova BioProfile 400 (Nova Biomedical, Waltham, MA, USA). The kinetics were calculated based on the following equations:

Specific glutamine consumption rate:

(1) $$Q_{g\ln}=\frac{S_{1}-S_{2}}{\int_{t_{1}}^{t_{2}}Nf(t)dt}$$

Specific ammonia production rate:

(2) $$q_{{\text{NH}}_{4}^{+}}=\frac{P_{2}-P_{1}}{\int_{t_{1}}^{t_{2}}Nf(t)dt}$$

where *Q_gln_* and *q*_NH4_^+^ was the specific glutamine consumption rate and the specific ammonia production rate of cells, *S_1_* and *P_1_* were the concentration of glutamine and ammonia at the time point of *t_1_*,* S_2_* and *P_2_* were the concentration of glutamine and ammonia at the time point of *t_2_*. $\int_{t_{1}}^{t_{2}}Nf(t)dt$ was the time integral of viable cell number and *f(t)* was fitted to the cell density.

### 2.7 Nutrient transporters expression

Surface GLUT1 and CD98 expression gated on CD3^+^ cells were examined by binding to the GLUT1 ligand fused to GFP (Metafora Biosystems, Evry cedex, France) and PE‐conjugated anti‐human CD98 antibody (BD Bioscience), respectively, and analysed using a FACS Aria I cytometer (BD Bioscience) and/or a ImageStream^X^ Mark II imaging flow cytometer (Merck) on FITC and PE channel.

The expression of ASCT2, SNAT1 and SNAT2 were measured by Western blotting. For protein extraction, expanded CIK cells for 7 days or fresh isolated CD3^+^cells were lysed in radioimmune precipitation assay protein extraction buffer (Beyotime, Shanghai, China) supplemented with protease inhibitor mixture (Beyotime) for 30 minutes on ice. After homogenization, samples were centrifuged at 12 000 × *g* for 15 minutes. Total soluble proteins from the supernants were measured using a BCA Protein Assay Kit (Beyotime). Equivalent protein concentrations were loaded on SDS‐PAGE gels (EpiZyme, Shanghai, China) and probed with primary Abs, rabbit anti‐ASCT2 (Cell Signaling Technology, Danvers, MA, USA), rabbit anti‐SNAT1 (Cell Signaling Technology), rabbit anti‐SNAT2 (Abcam, Cambridge, MA, USA), and mouse anti‐actin (Cell Signaling Technology). Secondary Abs anti‐mouse HRP (Cell Signaling Technology) and anti‐rabbit HRP (Signalway Antibody, College Park, MD, USA) were followed by Immobilon Western Chemiluminescent HRP Substrate (Millipore, Darmstadt, Germany) for visualization.

### 2.8 Enzyme activity

Expanded CIK cells for 7 days or fresh isolated CD3^+^ cells were analysed for the enzyme activities of PFK, G6PDH, GLS and GDH according to the manufacturers’ instruction. All enzyme activity detection assays were purchased from Comin Biotechnology (Suzhou, China).

### 2.9 Intracellular metabolites

Cells were collected at indicated time and analysed for intracellular ATP, NADP, NADPH levels according to the manufacturers’ instruction using ATP Assay Kits (Beyotime) and Amplite™ Colorimetric NADP/NADPH Ratio Assay Kits (AAT Bioquest, Sunnyvale, CA, USA), respectively.

### 2.10 Extracellular flux analysis

Extracellular flux analysis was carried on using a Seahorse XF96 analyser (Agilent Lexington, MA, USA).^33^, ^46^ 2 × 10^5^ expanded CIK cells in static and dynamic cultures for 7 days or freshed isolated CD3^+^ cells were seeded in plates coated with Cell‐Tak (Corning). After 1 hour, the plate was loaded into the instrument to determine oxygen consumption rate (OCR) and extracellular acidification rate (ECAR). For glycolytic stress tests, cells were plated in glucose‐free assay medium. During the course of the assay, cultures were injected with 10 mmol/L glucose, 2 µmol/L oligomycin and 50 mmol/L 2‐DG. For the mitochondrial stress tests, cells were plated in assay medium containing 1 mmol/L pyruvate, 2 mmol/L glutamine and 10 mmol/L glucose. During the course of the assay, cultures were injected with 2 µmol/L oligomycin, 0.5 µmol/L FCCP and 0.5 µmol/L rotenone/antimycin A. All reagents here were purchased from Agilent.

### 2.11 CFD modelling

Oxygen mass transfer coefficient (*k_L_*) modelling and numerical strategies were according to previous work of Li et al.^47^ The flow field formed in flasks was compared under the condition with filling volume of 30 mL and rotating speed of 0 or 130 rpm. The equations in CFD model are numerically solved by the commercial software package CFX 11.0 (ANSYS Inc., Canonsburg, PA, USA).

### 2.12 Statistics

Values were presented as mean ± standard error. Student's *t* test or one‐way ANOVA was applied to evaluate the significance of differences. *P < *0.05 was considered as statistically significant.

## 3 RESULTS

### 3.1 Dynamic cultures favoured better ex vivo expansion of CIK cells with cytotoxicity

**Figure 1:**
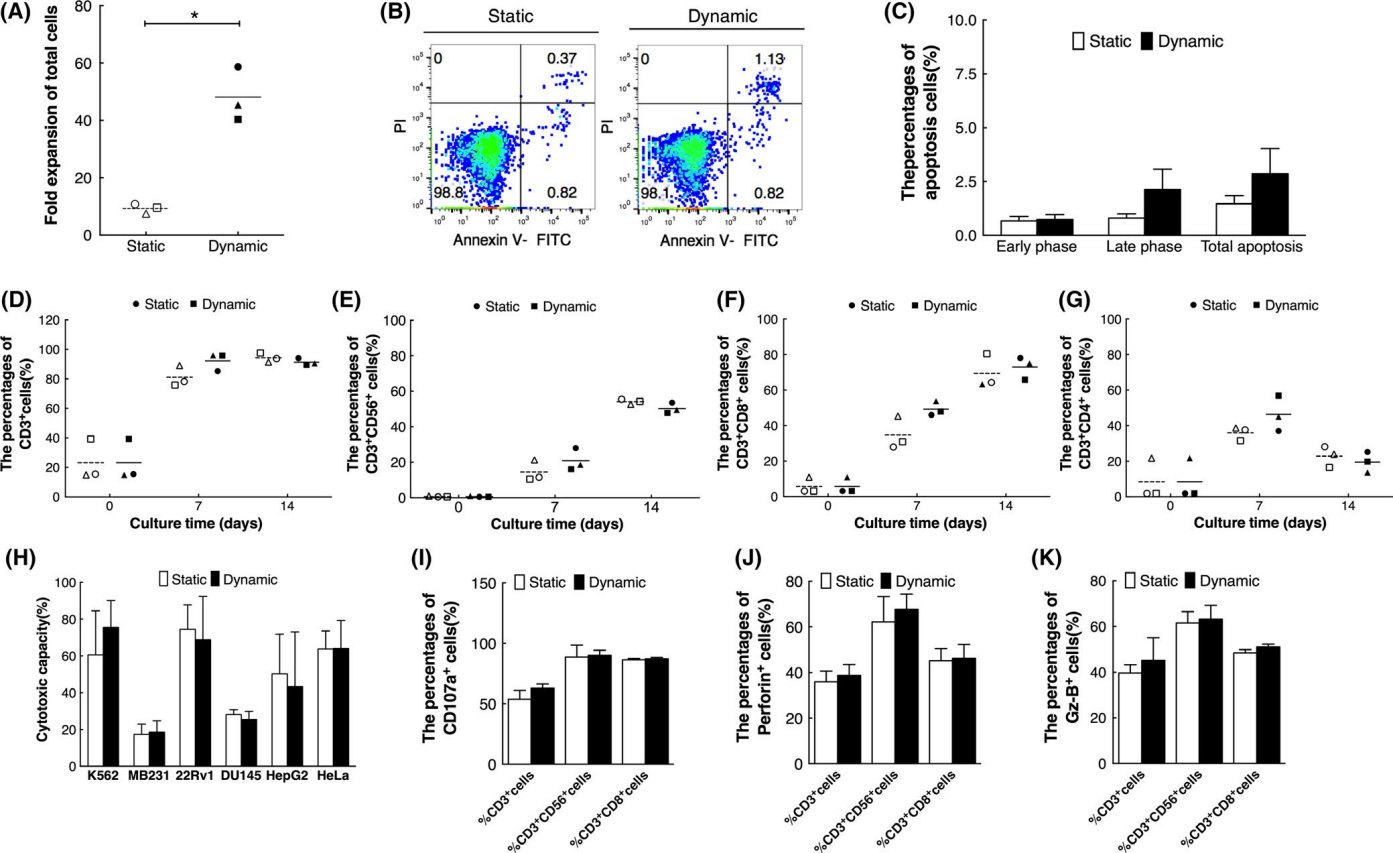
Ex vivo expansion characters of CIK cells in static and dynamic cultures (n = 3). A, Fold expansion of total cells on day 14. B, Representative flow cytometry analysis of cell apoptosis. C, Percentages of apoptotic cells in cultures on day 14. D, Percentages of CD3^+^ cells. E, Percentages of CD3^+^CD56^+^ cells. F, Percentages of CD3^+^CD8^+^ cells. G, Percentages of CD3^+^CD4^+^ cells. H, Cytotoxic capacity of expanded CIK cells on day 14 at the E: T ratio of 10:1. I, Percentages of CD107a^+^ cells. J, Percentages of perforin^+^ cells. K, Percentages of Gz‐B^+^ cells. Each hollow or black symbol represents one independent sample of static or dynamic cultures, dashed lines and solid lines represent mean values. *Compared with static cultures, *P < *0.05

Ex vivo expansion characters of cells in static and dynamic cultures were analysed and shown in Figure 1. After a 14‐day culture, the expansion folds of total cells in dynamic cultures were 48.14 ± 9.47 folds, significantly higher than the 9.29 ± 1.69 folds in static cultures (Figure 1A, *P < *0.05). On day 14, the percentages of total apoptotic cells in two cultures were lower than 5%, no significant difference was found between the two cultures (Figure 1B,C, *P* > 0.05). Further, the percentages of CD3^+^ cells, CD3^+^CD56^+^ cells, CD3^+^CD8^+^ cells and CD3^+^CD4^+^ cells in static and dynamic cultures were comparable (Figure 1D‐G, *P* > 0.05). Furthermore, cells in dynamic cultures had a similar broad‐spectrum cytotoxicity as cells in static cultures (Figure 1H‐K, *P* > 0.05). These results indicated that dynamic cultures favoured better ex vivo expansion of CIK cells without impairing their cell phenotype and physiologic functions.

### 3.2 Dynamic cultures provide a better mass transfer environment

**Figure 2:**
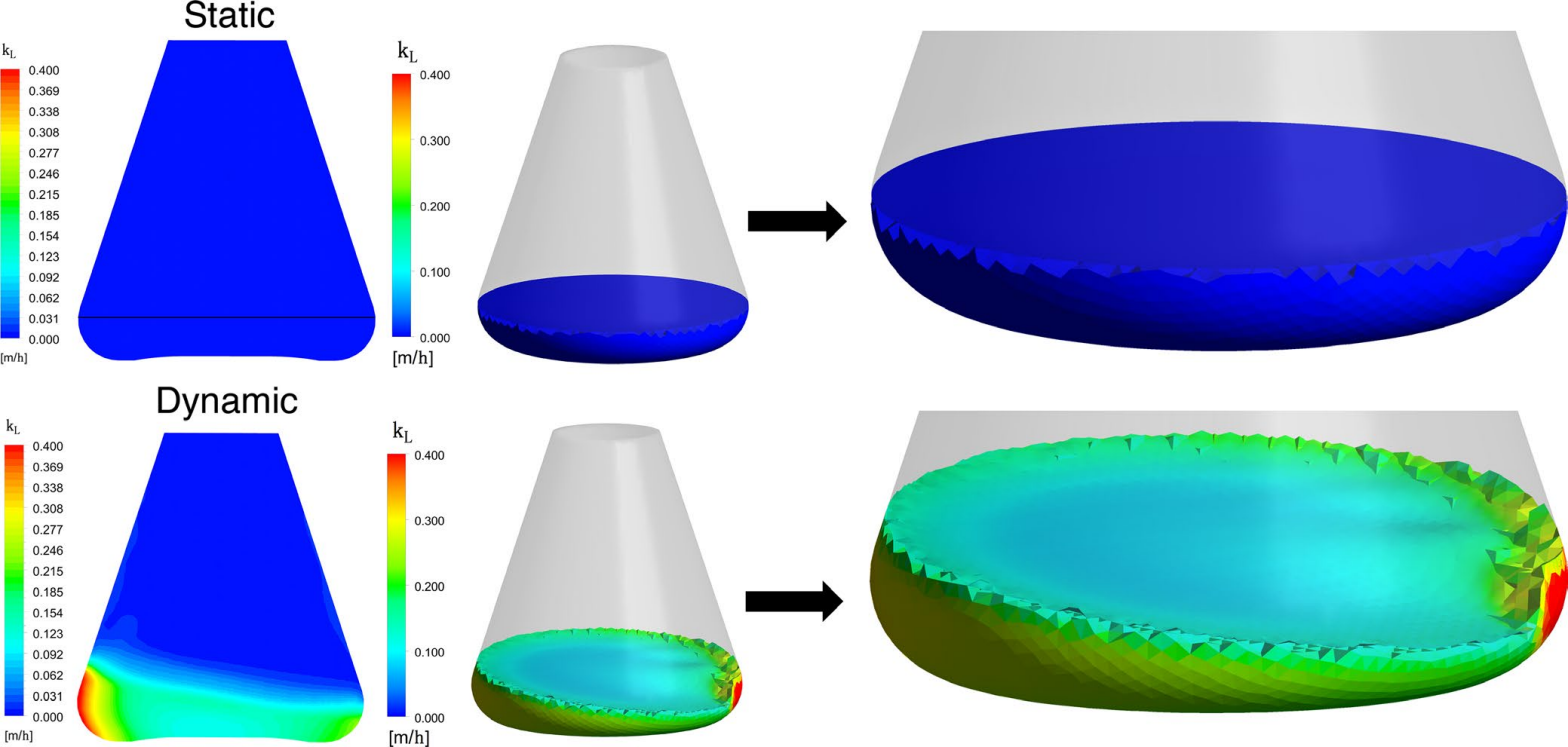
Computational fluid dynamics modelling of oxygen mass transfer coefficient (*k_L_*) in static and dynamic cultures

Oxygen in the culture environment is an important factor for cell growth and proliferation. Through CFD modelling of *k_L_*, the mass transfer differences could be easily observed. As shown in Figure 2, *k_L_* was obviously higher in dynamic cultures, illustrating that dynamic cultures enhanced oxygen transfer efficiency and could supply more oxygen into the microenvironment which were beneficial for CIK cell proliferation.

### 3.3 Enhanced surface GLUT1 expression and glycolytic enzyme activity results in high‐glucose uptake activity of cells in dynamic cultures

Different metabolism patterns of CD3^+^ cells were adopted before and after cytokines stimulation, and cell metabolism patterns were closely associated with cell proliferation^31^; hence, the glucose metabolism was first investigated to understand the relationship between cell metabolism and expansion during the ex vivo expansion of CIK cells.

**Figure 3:**
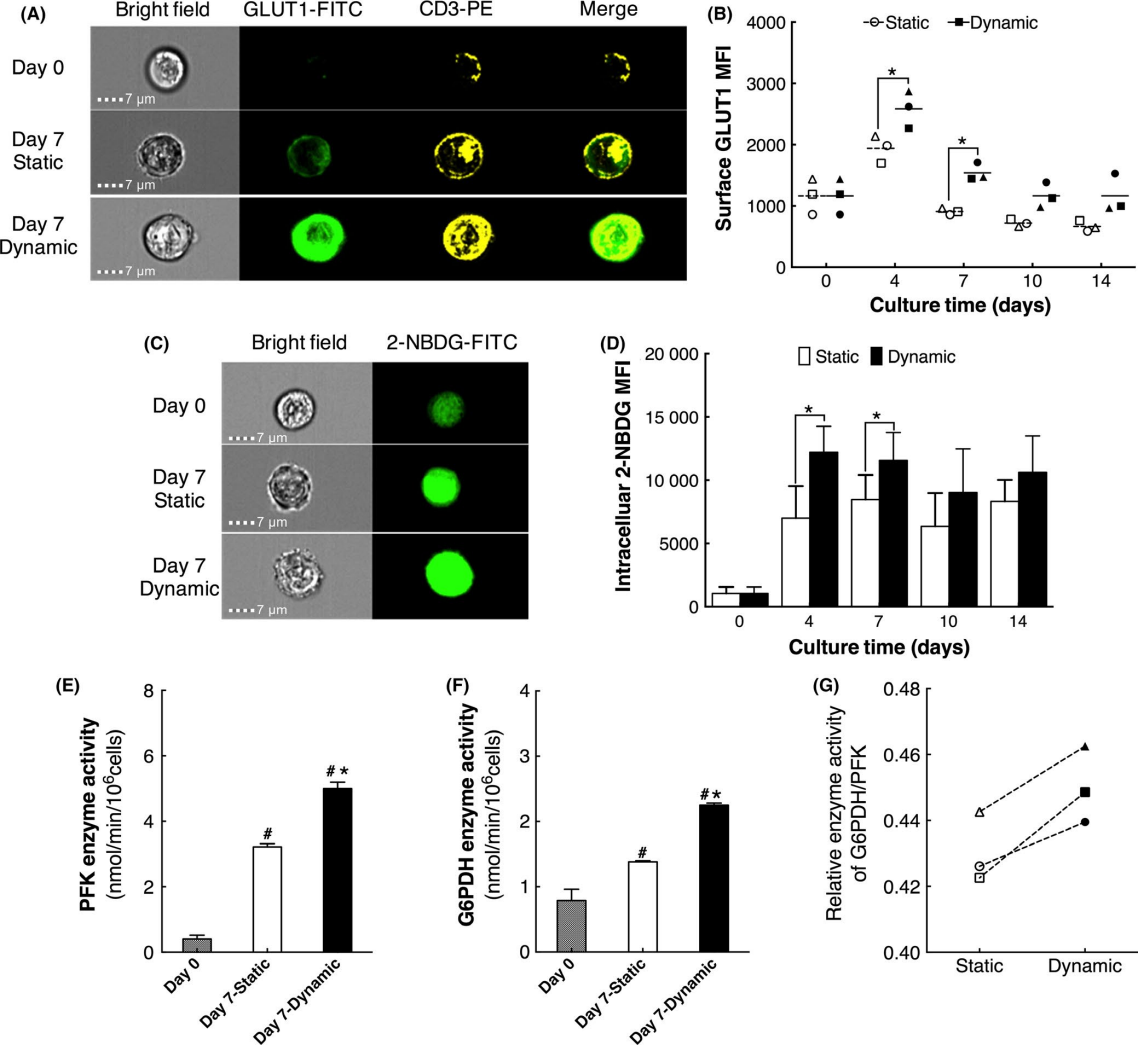
Glucose metabolism of cells cultured in static and dynamic cultures (n = 3). A, Multispectral single and merged images. B, Surface GLUT1 MFI of cells gated on CD3^+^ cells. C, Multispectral single and merged images. D, The 2‐NBDG MFI of cells. E, PFK activity. F, G6PDH activity. G, Relative G6PDH/PFK activity. Each hollow or black symbol represents one independent sample of static or dynamic cultures, dashed lines and solid lines represent mean values. #Compared with day 0, *P < *0.05; *Compared with static cultures, *P < *0.05. G6PDH, glucose‐6‐phosphate dehydrogenase; PFK, phosphofructokinase

Glucose was absorbed into cells mainly via GLUT1 in human CD3^+^ cells. Since the main cell population was CD3^+^ cells in ex vivo CIK cultures, the expression of surface GLUT1 gated on CD3^+^ cells was examined by flow cytometry (Figure 3A). The results showed that surface GLUT1 expression of cells in dynamic cultures was significantly higher than that of cells in static cultures during the culture process (Figure 3B, *P < *0.05), which may suggest the higher glucose uptake ability of cells in dynamic cultures.

Then, the glucose uptake activity of cells in static and dynamic cultures was validated by a 2‐NBDG assay (Figure 3C). During the culture process, intracellular 2‐NBDG was significantly increased on day 4 and decreased gradually after day 7. However, compared with static cultures, 2‐NBDG in cells of dynamic cultures was significantly higher (Figure 3D, *P < *0.05). These results demonstrated that cells in dynamic cultures possessed higher glucose uptake activity via the upregulation of surface GLUT1 expression.

Upon entering cells, glucose would be immediately phosphorylated to G6P for glycolysis or PPP. Therefore, the activities of PFK and G6PDH, two key rate‐determining enzymes in glycolysis and PPP were determined, respectively. The PFK and G6PDH activities of fresh isolated CD3^+^ cells were 0.40 ± 0.12 nmol/min/10^6^ cells and 0.79 ± 0.17 nmol/min/10^6^ cells, respectively. After ex vivo expansion, the PFK and G6PDH activities of cells cultured in static and dynamic cultures for 7 days were both increased significantly (Figure 3E,F, *P < *0.05). Moreover, compared with cells in static cultures, cells in dynamic cultures had observably higher PFK and G6PDH activities (Figure 3E‐G, *P < *0.05). Consequently, these results may indicate that the glucose metabolic flux in cells of dynamic cultures were both enhanced by the upregulation of PFK and G6PDH activities.

### 3.4 Upregulated glutamine consumption also contributes for better cell expansion in dynamic cultures

In addition to glucose, glutamine is another important carbon and energy source for cell proliferation. Glutamine also depends on transporters to enter cells. Therefore, four important glutamine transporters of cells in static and dynamic cultures were analysed.

**Figure 4:**
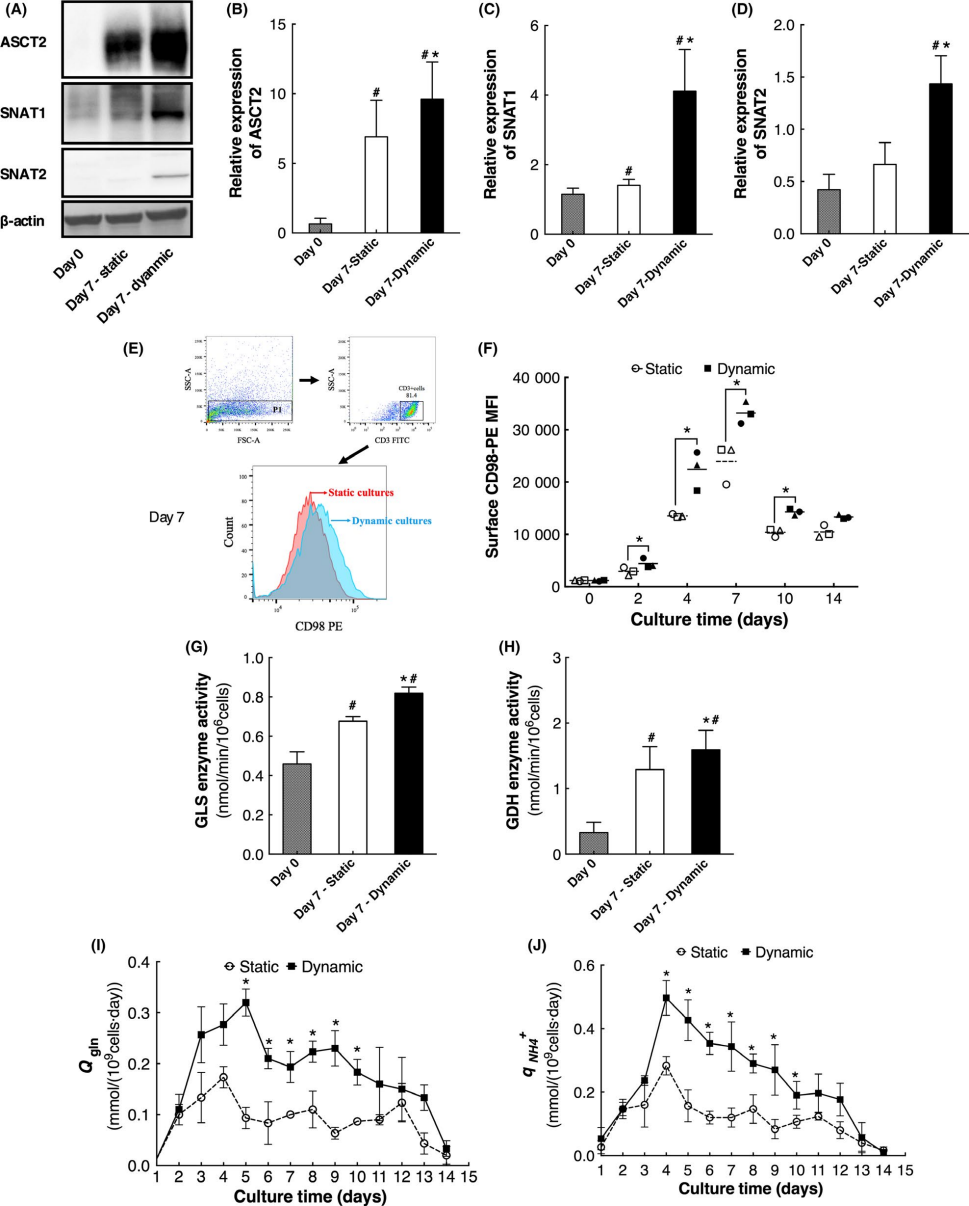
Glutamine metabolism of cells in static and dynamic cultures (n = 3). A, ASTC2, SNAT1 and SNAT2 were determined by Western blotting. B, Relative expression of ASTC2. C, Relative expression of SNAT1. D, Relative expression of SNAT2. E, Representative flow cytometric analysis of surface CD98 expression, red and blue line represent static and dynamic cultures, respectively. F, The surface CD98‐PE MFI of cells. G, GLS activity. H, GDH activity. I, *Q_gln_*. J, *q*_NH4_^+^. Each hollow or black symbol represents one independent sample of static or dynamic cultures, dashed lines and solid lines represent mean values. #Compared with day 0, *P < *0.05; *Compared with static cultures, *P < *0.05

The expression of ASCT2, SNAT1 and SNAT2 were analysed by Western blotting (Figure 4A). Compared with fresh isolated cells, the expression of all these three transporters was enhanced in cultured cells (*P* < 0.05). And the expression of ASCT2, SNAT1 and SNAT2 in cells of dynamic cultures were significantly higher than those of cells in static cultures (Figure 4B‐D, *P < *0.05). Moreover, since CD98 is a subset of the CD98/LAT1 heterodimeric amino acid transporter, the expression of this heterodimer was analysed by the expression of surface CD98. During the culture process, surface CD98 expression of cells in dynamic cultures was significantly higher than those of cells in static cultures (Figure 4E,F, *P < *0.05), indicating the expression of the CD98/LAT1 heterodimeric transporter was also enhanced in cells of dynamic cultures.

Further, the activities of key enzymes in glutaminolysis, GLS and GDH, were determined. The GLS and GDH activities of cells in static and dynamic cultures were significantly higher than those of fresh isolated CD3^+^ cells (Figure 4G,H, *P < *0.05). Meanwhile, GLS and GDH activities of cells in dynamic cultures were significantly higher than those of cells in static cultures (Figure 4G,H, *P < *0.05), suggesting that the potential metabolic flux of glutaminolysis may be enhanced in cells of dynamic cultures.

Furthermore, the *Q_gln_* and *q*_NH4_^+^ of cells in static and dynamic cultures were calculated by Equations (1) and (2) and shown in Figure 4I and J. The *Q_gln_* and *q*_NH4_^+^ of cells in dynamic cultures were both significantly higher than those of cells in static cultures (*P < *0.05), illustrating the upregulation of glutaminolysis metabolic rate. These results demonstrated that cells in dynamic cultures enhanced glutamine consumption through upregulating the expression of glutamine transporters and activities of GLS and GDH for producing more intermediates that are consumed by biosynthetic processes, supporting the better cell expansion.

### 3.5 Improved ATP production ability accounts for high cell proliferation ability in dynamic cultures

**Figure 5:**
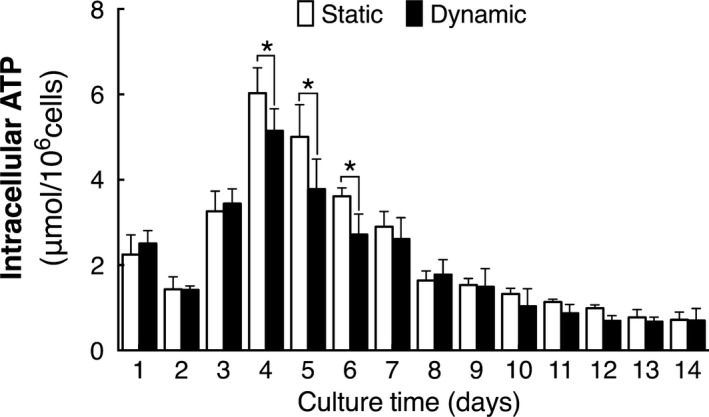
Intracellular ATP level of cells in static and dynamic cultures (n = 3). *Compared with static cultures, *P < *0.05

For proliferation, large amounts of ATP would be needed for the synthesize of biomass, while glucose and glutamine are two major substances of energy source. Considering both glucose and glutamine consumption were enhanced, intracellular ATP content was measured to investigate the energy metabolism of cells in two cultures. As shown in Figure 5, ATP content in cells of both cultures ascended first then descended, surprisingly, it was found that ATP content in cells of dynamic cultures were similar or a bit lower than those of cells in static cultures (*P < *0.05).

**Figure 6:**
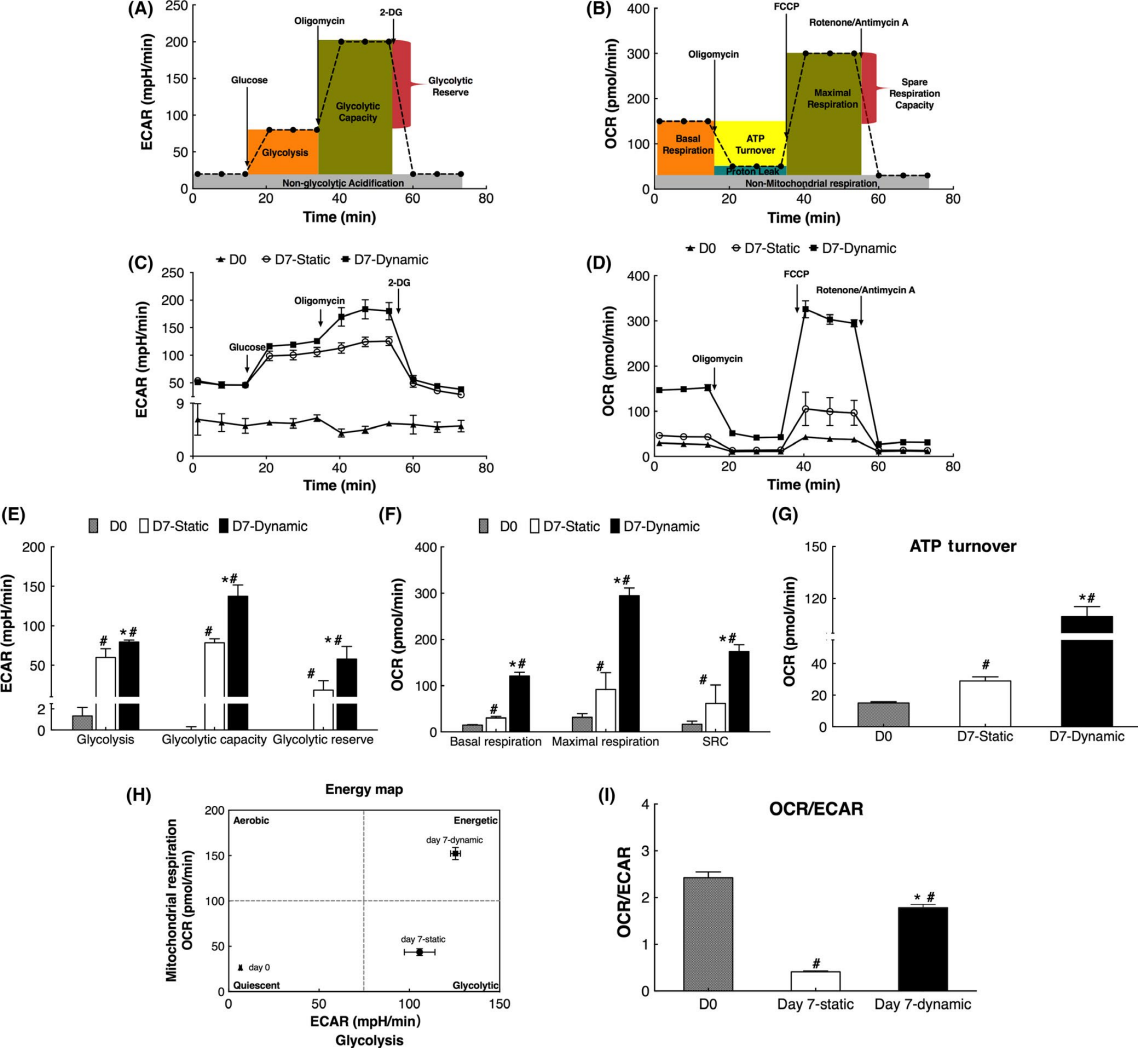
The extracellular acidification rate (ECAR) and oxygen consumption rate (OCR) of cells cultured in static and dynamic cultures (n = 3). A, Diagram of glycolytic function. B, Diagram of mitochondrial function. C, Glycolytic stress tests. D, Mitochondria stress tests. E, Basal ECAR, glycolytic capacity and glycolytic reserve of cells. F, Basal OCR, maximal respiration and spare respiration capacity of cells. G, ATP turnover. H, Energy map. I, OCR/ECAR ratio. #Compared with day 0, *P < *0.05; *Compared with static cultures, *P < *0.05

Since intracellular ATP level was a result of generation and consumption, we measured ECAR (an indicator of aerobic glycolysis) and OCR (an indicator of OXPHOS) to investigate the ATP production ability of cells in two cultures (Figure 6A‐D). The results showed that compared with fresh isolated CD3^+^ cells, all ECAR and OCR values were significantly higher in expanded CIK cells (Figure 6E‐G, *P < *0.05), demonstrating expanded CIK cells went into a more vigorous metabolic state (Figure 6H).

In addition, all ECAR and OCR values in expanded CIK cells from dynamic cultures were found significantly higher than those from static cultures (Figure 6E‐G, *P < *0.05). The higher ECAR and OCR values indicated that expanded CIK cells in dynamic cultures were in a metabolically higher energy state (Figure 6H). The ratio of OCR/ECAR was higher in dynamic cultures than in static cultures (Figure 6I, *P < *0.05), indicating that expanded CIK cells were in a higher energy state in dynamic cultures through mechanisms that relied more on mitochondrial metabolism than on glycolysis.

Collectively, these results showed that the two major ATP‐generating metabolic ways, glycolysis and OXPHOS, were both enhanced in cells of dynamic cultures. More importantly, cells in dynamic cultures were more oxidative to generate ATP which was needed for the production of biomass.

**Figure 7:**
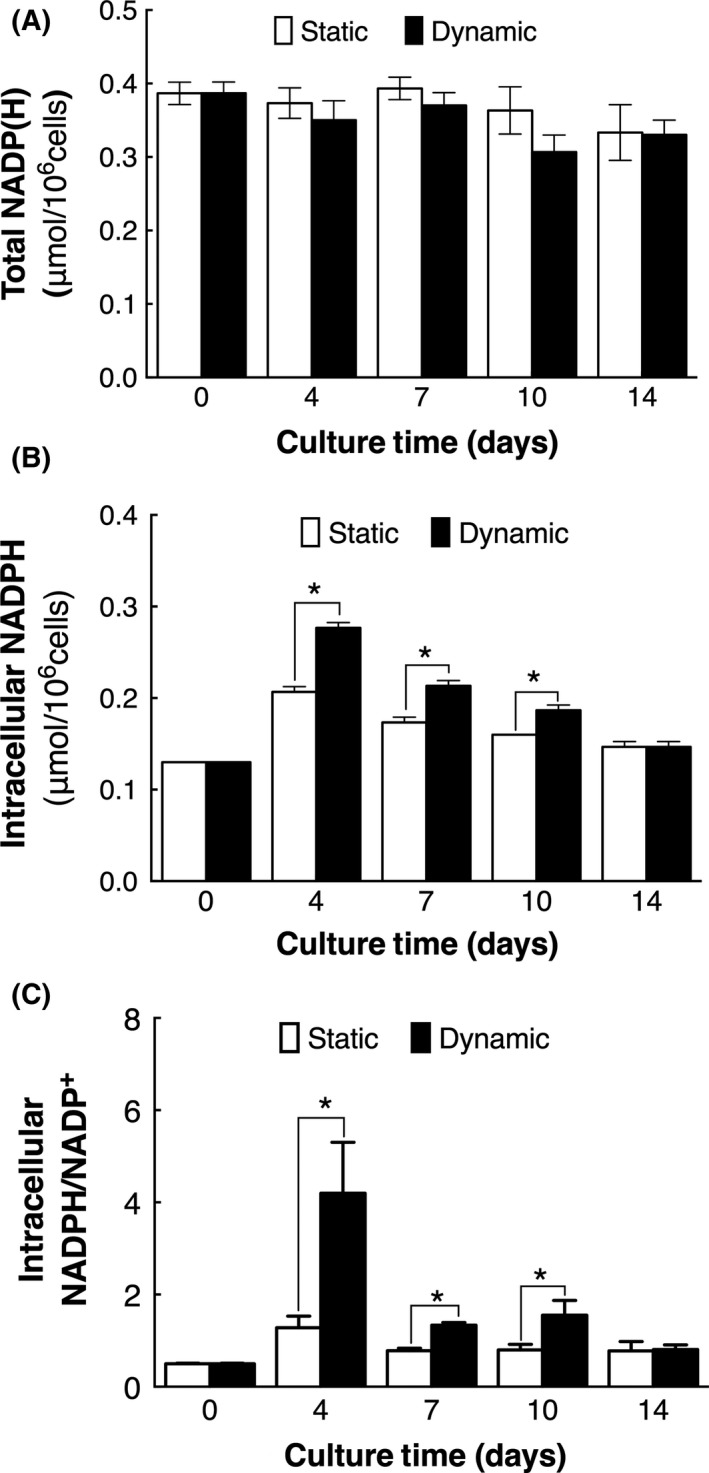
Intracellular NADP(H) level of cells cultured in static and dynamic cultures (n = 3). A, Total NADP(H). B, NADPH. C, NADPH/NADP^+^ ratio. *Compared with static cultures, *P < *0.05

In addition, NADPH was another important cofactor in T‐cell activation, differentiation and proliferation.^48^ Thus, intracellular NADP(H) were determined and shown in Figure 7. Total NADP(H) (NADP^+^ and NADPH) in cells of two cultures maintained similar during the whole culture process (Figure 7A), while intracellular NADPH and NADPH/NADP^+^ ratio increased after expansion, notably. NADPH and NADPH/NADP^+^ ratio in cells of dynamic cultures were found significantly higher than those in cells of static cultures (Figure 7B,C, *P < *0.05). In consideration of that NADPH is mainly produced through PPP, it could be deduced that the metabolic flux of PPP in cells of dynamic culture was accelerated, which was consistent with the results of enhanced G6PDH activity.

## 4 DISCUSSION

Immune effector cells‐based immunotherapy has become a reality for many diseases.^19^ Many strategies had been developed to optimize the ex vivo expansion of immune effector cells for producing cell products effectively for clinical treatments. In our previous study, it was demonstrated that dynamic cultures could improve the ex vivo expansion of CIK cells without impairing the cytotoxicity of expanded CIK cells,^45^ which were further demonstrated in the present study.

It was reported that only cells which were fully activated could undergo proliferation.^49^, ^50^ Thus, cell activation played key roles in the ex vivo expansion of CIK cells. Our results showed that after cytokines stimulation, cells in dynamic cultures had higher glucose and glutamine consumption via inducing higher expression of nutrient transporters and activities of metabolic enzymes. These results may be related to the higher activation state of cells in dynamic cultures which were previously found.^45^ It had been reported that cell activation could be affected by cytokines,^51^ cell density,^52^ oxygen tension,^53^, ^54^ etc. Therefore, it would be important to provide a suitable culture environment for cells to expand more efficiently. Dynamic cultures not only provided a more homogeneous environment to increase the cell‐to‐cell and cell‐to‐cytokines contact, but also improved oxygen mass transfer to increase the oxygen tension in the microenvironment, which was beneficial for cell activation. Therefore, it was reasonable to infer that dynamic cultures improved cell expansion via regulating cell activation and cell activation‐induced metabolism reprogramming.

Further, it had been noted that metabolism patterns of lymphocytes after cell activation was correlated with the cell proliferation ability.^33^ Our results showed that cells in dynamic cultures elevated both glycolysis metabolic rate via upregulating GLUT1expression and PFK activities, which was further demonstrated by 2‐NBDG assays and higher ECAR values. This results were consistent with the results of our previous study.^45^ Meanwhile, the PPP metabolic rate was also elevated via upregulating G6PDH activities, resulting higher intracellular NADPH level. In addition to glucose, higher glutamine consumption rate of cells was observed in dynamic cultures, which could support T cells for the synthesis of protein, nucleotides and amino sugars, all of which were important for proliferating T cells.^28^, ^55^ Moreover, the oxidation of glutamine in TCA cycle could produce ATP, potentially allowing activated T cells to divert glucose to other biosynthetic pathways.

ATP is a key donor that provides energy for cellular processes of CD3^+^ cells, and it can be produced mainly via two pathways, glycolysis and TCA cycle/OXPHOS.^30^ In this study, lower ATP content was found in cells of dynamic cultures. The ATP production ability of cells in dynamic cultures was demonstrated to be improved by the enhanced ECAR and OCR of cells, which were indicators of glycolysis and OXPHOS, respectively. So, the decreased cellular ATP content may result from more consumption for cell proliferation.

In addition, cells in dynamic cultures were more energetic and oxidative with a higher basal OCR value and OCR/ECAR ratio. More importantly, the oxidative ATP turnover was found much higher in cells of dynamic cultures, which meant cells in dynamic cultures consumed more oxygen for ATP production via OXPHOS during ex vivo expansion. Meanwhile, dynamic cultures provided a better mass transfer which could supply more oxygen into the microenvironment, resulting in an upregulated OCR of expanded CIK cells for better ATP biosynthesis. These findings together may account for the accelerated cell growth in dynamic cultures.

**Figure 8:**
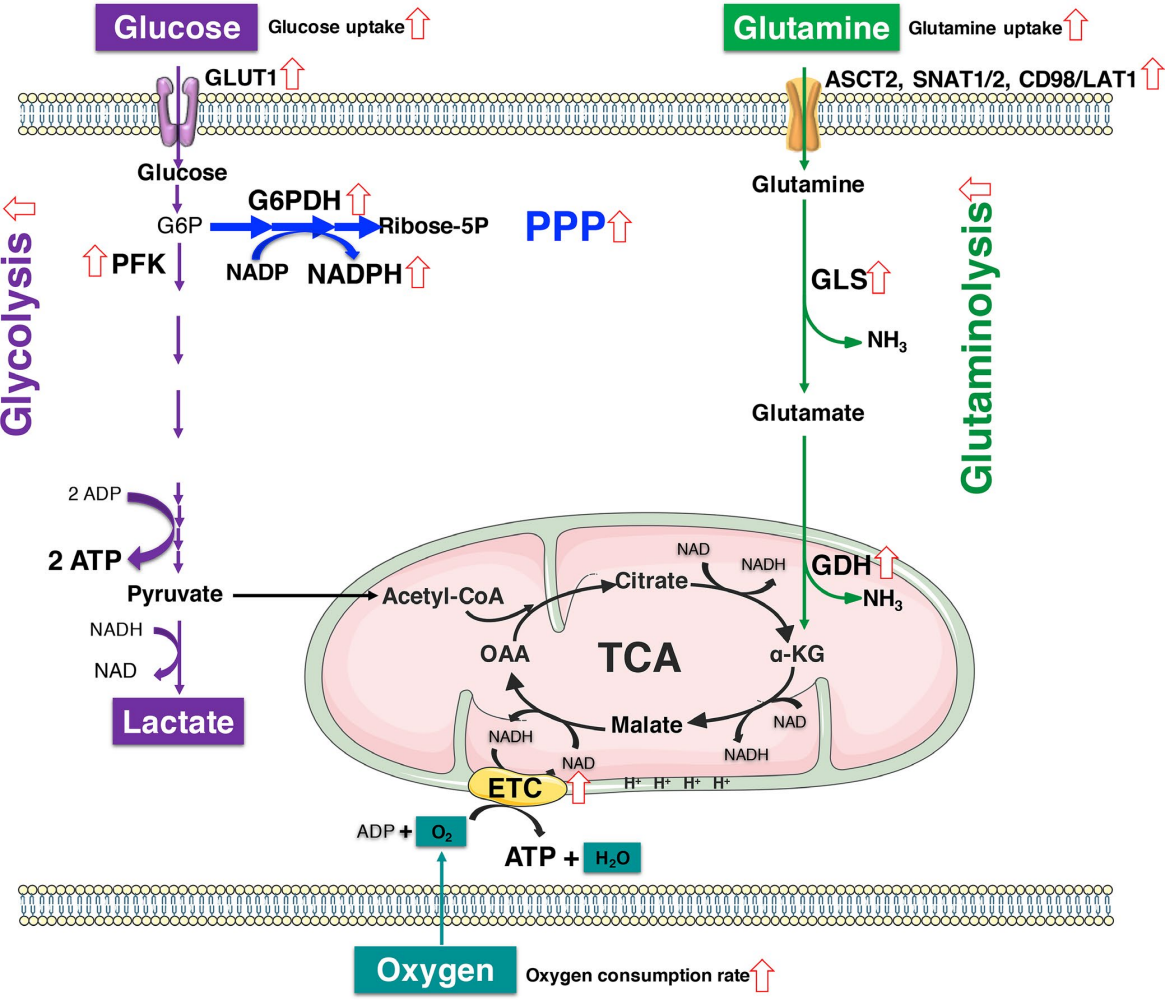
Elevated overall metabolic activities of cells in dynamic cultures. Upregulated transporters, enzyme activities, or metabolites were indicated with red arrows

In conclusion, dynamic cultures enhanced oxygen transfer efficiency, accelerated glucose and glutamine consumption by enhancing the expression of relative transporters and the activities of key metabolic enzymes. Dynamic cultures enhanced overall metabolic activities of cells for ATP production and elevated metabolic flux via PPP for better cell expansion (Figure 8). These findings provide a valuable guidance for scale‐up ex vivo expansion of CIK cells and other immune effector cells.

## CONFLICT OF INTEREST

The authors declare that they have no conflicts of interest with the contents of this article.
